# Tunnel Vision: A Novel Approach for Blood Clot Retrieval Using Cryotherapy through a Flexible Bronchoscope

**DOI:** 10.1155/2023/6620825

**Published:** 2023-11-11

**Authors:** Fernando Figueroa Rodriguez, Parminder Minhas, Houssein A. Youness

**Affiliations:** ^1^Interventional Pulmonary Program, Section of Pulmonary, Critical Care and Sleep Medicine, The University of Oklahoma Health Sciences Center, USA; ^2^The Oklahoma City Veteran Affairs Medical Center, USA

## Abstract

Cryoprobes inserted through a bronchoscope in the airways have frequently been used for the removal of foreign bodies and clots. We present a case of a 72-year-old man who presented with COVID-19 pneumonia and respiratory failure, requiring intubation and proning. During his stay, he developed pneumothoraces in the left hemithorax, which necessitated the placement of two large-bore chest tubes. However, the pneumothorax recurred. There was no air leak in either chest tube, and attempts to restore their patency through flushing or suctioning were unsuccessful. A disposable flexible bronchoscope was inserted into the chest tubes, allowing visualization of the source of occlusion and identification of a blood clot obstructing both tubes. These clots were successfully removed using a cryoprobe inserted through the working channel of the bronchoscope, leading to the restoration of chest tube patency and resolution of the pneumothorax.

## 1. Introduction

Cryoprobes, inserted through bronchoscopes, have been utilized for cryoablation and cryoextraction of abnormal tissue in the airway [1]. Their effectiveness has been documented in cases of benign and malignant airway obstruction, as well as in pleural diseases, with several reports highlighting the successful use of cryotherapy for retrieving tracheobronchial blood clots [2–5]. However, there are no prior descriptions of employing this intervention for the removal of blood clots from thoracostomy tubes. Here, we present a unique case in which chest tube patency was restored through blood clot extraction using a cryoprobe inserted via a flexible bronchoscope in a critically ill patient with recurrent pneumothorax.

## 2. Case Presentation

A 72-year-old male presented to the emergency room with hypoxemic respiratory failure related to COVID-19 pneumonia. He had a previous medical history of hypertension, obstructive sleep apnea, renal cell carcinoma status post nephrectomy, and morbid obesity. Treatment was initiated with dexamethasone and remdesivir. However, due to worsening hypoxemia secondary to adult respiratory distress syndrome (ARDS), the patient required intubation on day two, and proning therapy was initiated using a Rotoprone bed. Additionally, a heparin drip was administered for suspected pulmonary emboli.

**Figure 1:**
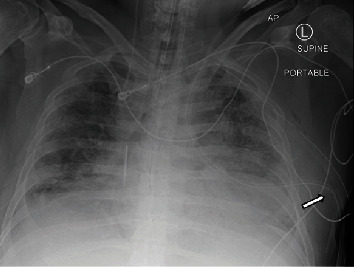
Last available preintervention chest X-ray showing kinking of chest tube #1 in the subcutaneous tissue (arrow).

On day four, the patient developed a left-sided pneumothorax, necessitating the placement of a 28 French chest tube (chest tube #1) to achieve lung reexpansion. On day 16, the patient experienced acute worsening with oxygen desaturation, raising suspicion of pneumothorax recurrence, as there was no air leak from chest tube #1. The last available CXR (Figure 1) prior to the desaturation event revealed subcutaneous kinking of the chest tube. However, bedside examination showed no external kinking, and attempts to straighten the chest tube did not improve the air leak. Further investigation revealed occlusion of the chest tube with clots that could not be flushed. Subsequently, a second 28 French chest tube (chest tube #2) was placed, leading to improved oxygen desaturation.

On day 17, the on-call physician was urgently called to the bedside due to oxygen desaturation (70%) while the patient was in the prone position on 100% FiO2. At that time, there was no noticeable air leak in either tube. A bedside ultrasound examination revealed absent lung sliding on the left side, indicating a potential recurrent pneumothorax. Examination of chest tube #2 unveiled a large blood clot, but attempts to remove the obstruction using a sterile suction catheter were unsuccessful.

**Figure 2:**
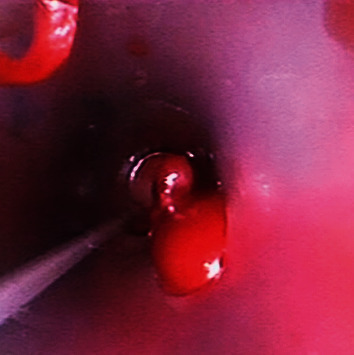
Interior of chest tube #2 showing that the lumen is distally occluded by a blood clot.

**Figure 3:**
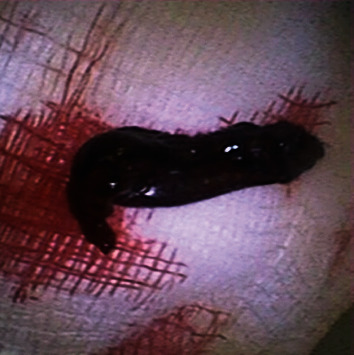
The blood clot extracted in its entirety from chest tube #2.

**Figure 4:**
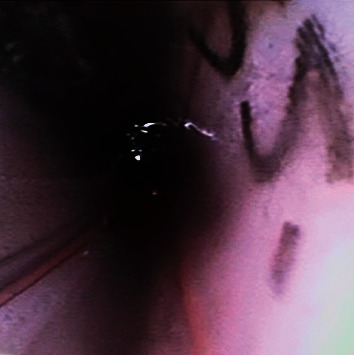
Interior of chest tube #2 showing patency restored.

**Figure 5:**
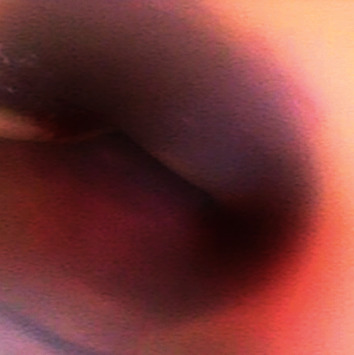
Chest tube #1 shows a kink at the level of the intercostal space.

**Figure 6:**
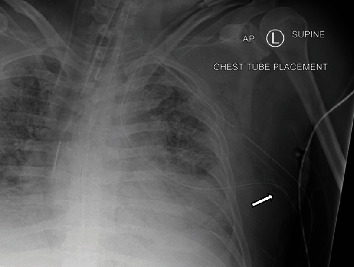
Postintervention chest X-ray showing improved kinking in chest tube #1 and fully expended lung in a patient with COVID-19 respiratory failure on mechanical ventilation.

Given the experience with cryotherapy, a decision was made to employ a cryoprobe for blood clot retrieval. A flexible disposable bronchoscope (Ambu Scope 4 Bronchoscope Large 5 mm/2.8 mm) was inserted into chest tube #2 to visualize the blood clot (Figure 2). A 2.4 mm Erbe cryoprobe was then inserted through the working channel of the bronchoscope and carefully placed within the blood clot. A five-second freeze time was applied, followed by the successful removal of the blood clot (Figure 3), thereby restoring patency to chest tube #2 (Figure 4). The bronchoscope was subsequently inserted into chest tube #1, revealing a kink that hindered drainage (Figure 5). By placing a roll of gauze bandage under the insertion site and reapplying the dressing, the kink was resolved. Continuing the bronchoscope's distal advancement, a blood clot was visualized in chest tube #1. Employing the previously described technique, the blood clot was extracted. As a result, air leaks were observed in both chest tubes, and the patient's oxygen saturation improved to 98%. Bedside lung ultrasound showed lung sliding, and a supine chest X-ray displayed expanded lungs with no pneumothorax (Figure 6). The heparin drip was discontinued.

## 3. Discussion

Thoracostomy tubes are widely used for draining fluid and air, as well as instilling medications such as fibrinolytic and sclerosing agents. However, complications can arise after insertion, including tube malposition, dislodgement, and blockage. The latter can lead to unresolved pleural collections or, in cases of pneumothorax, progress to life-threatening tension physiology [6].

Currently, there are no published standards for evacuating blood clots from chest tubes. Flushing the tube may be effective for small clots but is generally unhelpful for larger ones. The “milking or stripping” technique, involving maneuvers like squeezing or twisting the tube to create suction and dislodge the clot, is controversial and debatable in terms of efficacy, with potential harm due to negative pressure that may damage lung tissue [7]. The PleuraFlow Active Clearance Technology is a new system developed for sterile clot removal, but its use is limited to patients following cardiac surgery [8].

While the use of a flexible bronchoscope through a chest tube has been previously described for pleurisy evaluation [9], its employment for restoring tube patency has not been reported. The availability of disposable flexible bronchoscopes and cryoprobes makes it an acceptable approach for evaluating chest tube blockage and retrieving clots when identified. The traditional approach involves replacing the chest tubes, but in our patient, who already had two chest tubes and was in a prone position, placing a third tube posed additional challenges. Chest tube blockage can occur due to kinking or clot formation, both of which we were able to visualize and address in this case.

The mechanism of how the cryoprobe aids in clot removal relies on its cytoadherence, where the object of interest adheres to the cryoprobe due to the crystallization of water molecules at the interface. This object can then be extracted en bloc along with the probe, a process known as cryoextraction [10].

We hope that this case sheds light on safe troubleshooting for dysfunctional chest tubes, obviating the need for replacement, especially in patients with multiple comorbidities, existing multiple chest tubes, and in a prone position, as in our case. However, expertise in using this technique and the availability of sterile bronchoscopes and cryoprobes are essential for safely performing this procedure.

## Data Availability

Data sharing is not applicable to this case report, as no new data was created or analyzed in this study.
